# Medico-Legal

**Published:** 1908-09

**Authors:** E. S. McKee

**Affiliations:** Cincinnati


### Medico-Legal.

BY E. S. McKEE M. D., Cincinnati.

### LAWS CONCERNING CREMATION.

CREMATION and crime might go hand in hand were it not for stringent laws. An interesting case has occurred in England though there was fortunately no crime concealed. The wife of a medical man fell ill with measles complicated with bronchitis in the eighth month of her pregnancy. On the day of her death her husband with the consent of her brother, who was also a medical man, delivered the patient instrumentally in order to relieve the dyspnea from which she was suffering. The child was born dead and the mother died in a few hours but would have died sooner, the husband considered, but for the relief afforded by premature delivery. The undertaker was sent for and when told that it was desired that the body be cremated he produced the necessary form to be filled up. He was informed that the deceased had given birth to a stillborn child and he stated that the child could not be placed in the same coffin with the mother, and did in fact, later, bring to the house a second coffin for the reception of the body of the child. The father, however, expressed a wish that both bodies should be placed in one coffin. The undertaker superintended the preparation of the bodies, placed both of them in one shell and then removed them to the crematory where they underwent cremation. When the sides of the coffin fell away the superintendent noticed the body of a child lying at the feet of an adult corpse. For sentimental reasons action was brought against the undertaker. The prosecutor said that there seemed to be a want of frankness in the way in which the death certificate was made out. In a certificate from a medical man, which must be given before any body shall be cremated it was, he said, in the highest degree desirable that all the circumstances should be set out in regard to a death. As the deceased was suffering from two diseases—measles and bronchitis—it was clear that her death was accelerated by the death of a child, whereas the certificate made no reference whatever to that contributary cause of death. The husband when giving evidence said that when the defendant brought the coffin for the child he thought it better that both be cremated in one shell. He knew nothing about the regulations, nor had he any intention of deceiving the company or anything to do with drawing up or signing the certificates. There was no reason why, subject to proper certificates the two bodies might not have been cremated in one coffin. The court held that the responsibility of the defense had been proven and said that to mark the importance of the regulations being carried out literally and properly he assessed a fine and the costs amounting in the aggregate to about $75.00. It would be easy to criticise in this case but for sentimental reasons we will desist. Never having been placed in such a trying position as the husband and father and physician in this case it would hardly be fair to say what we would or would not have done under the circumstances.

---

### LENIENCY OF COURTS WITH INSURERS WHO ARE INACCURATE

In a case brought up recently the insured had been asked, "Have you any illness or infirmity" and "If you have any illness or infirmity have you fully recovered from it?" His answer was

"Had none." It appeared that ten years before the insured had had a miscarriage and required the use of instruments to complete delivery. The company disputed the liability on the ground that the answer was untrue. Although the company was held liable on other grounds the judge who tried the case expressed the opinion that the miscarriage was not an illness or infirmity and therefore the answer was not untrue. This decision is in accord with many older ones which go to show that the courts will not readily void a policy on the grounds that the insured has answered a purely medical question untruthfully. In a case mentioned in the *British Medical Journal,* March 4, 1905, an applicant was asked, "Have you ever met with an accident?" He plied "No." Whereas it was proven that he had fallen only a month before and had actually made a claim against an accident insurance company in respect to the injury so sustained. The court of appeals found that this was sufficient to vitiate the policy though the jury found, "the injury received was not of sufficient importance to need mentioning," and that "the answer was in substance true." A lady who had insured heavily had answered in the negative the question, "Have you at any time had brain fever or any other diseases of the brain?" She was later found dead with the remains of a bottle of poison by her side denoting suicide. The insurance company resisted payment on the grounds that she had concealed the fact that she had suffered an attack of acute mania following influenza. After treatment she recovered. She had been told that she was suffering from a nervous breakdown. The jury found that she had "foolishly not fraudulently" concealed the fact that she had had this illness. Judgment was given for the company but as no fraud was proven the premiums were ordered returned.

---

### DEATH UNDER CHLOROFORM AND ACCIDENT INSURANCE.

A TEAMSTER carrying accident insurance was injured during work the injury resulting in a dislocated hip-joint. He was put under chloroform and succumbed under the anesthesia. The insurance company refused to pay the insurance claiming that death was due to the anesthetic and not the accident. The courts decided adversely to the company, stating that the accident was responsible for the injury requiring the anesthetic, and that the insurance consequently covered death for this cause. The point was emphasised that an unsuccessful operation for conditions resulting from an accident does not release the insurance company from its responsibility.

### FALLEN IDOLS.

Having walked the wards of Saint Thomas's Hospital mid the ministrations of Florence Nightingale, sailed the Bosphorus and the Black Sea where her first victories were won, being proud to believe trained nurses, the Anglo Saxon variety at least, to be as pure as their spotless uniforms, or, as Shakespeare put it, "so dutious, diligent, so tender over her occasions, true, so neat, so nurse like." some recent developments are shocking. I once had a patient, a young woman, fair to look upon, who had served a term in each of three sporting houses. She had had the syphilis and gonorrhea and was lazy and trifling, without the first requisite for anything decent. Tiring of sporting she conceived the idea of taking up nursing. She had the unlimited gall to apply to be taken in at the private hospital of a medical friend of mine and gave me as reference. A letter came asking for recommendations. They were not produced. She was not taken in to this hospital to learn to be a nurse and I lost the bills she and her relatives owed me and lost them as patients. She applied and was admitted as a pupil in a New York Hospital. In a short period she will be a pretty pious nurse. Some day a weak and foolish man will fall under her care and in the silly stage of convalescence after a long illness, wealthy, perhaps, and somewhat advanced in years, yet not "too old to fawn upon a nurse," will propose in a fit of gratitude and be coyly accepted. I know, in fact, of another case where just this has happened. Let us hope that the man she gets will be just as bad as herself. I know of another case where a trained nurse seduced (advisedly) a handsome youth, afterward gave birth to twin boys, blamed them on the youth, got a divorce, married him, had him adopt the babies legally, and when proving unable to support them, left him and is now on the lookout for another victim. I know of another graduated trained nurse who is never so proud or happy as when she has succeeded in producing an abortion on herself, which, according to her statement, occurs about every other month, and who, if you would believe her has all the doctors she has nursed for, "on her list." Taking these startling facts into consideration it behooves schools for nurses to be more careful whom they admit to their classes. I am happy to say that these are, of course, very great exceptions to the average trained nurse.

